# Supplementary material for: The nature of the last universal common ancestor and its impact on the early Earth system
Source: Nat Ecol Evol. 2024 Jul 12;8(9):1654–66. doi: 10.1038/s41559-024-02461-1 (PMC11383801; doi:10.1038/s41559-024-02461-1)
Supplement: Supplementary file 1 — Supplementary Notes and Figs. 1–10. [file 41559_2024_2461_MOESM1_ESM.pdf]

nature ecology & evolution

Supplementary information

https://doi.org/10.1038/s41559-024-02461-1

The nature of the last universal common ancestor and its impact on the early Earth system

In the format provided by the authors and unedited

Supplementary Information:
The nature of the Last Universal Common Ancestor and its impact on the early Earth system

Notes
Reconciliation caveats
We summarise the evidence that genes were present at the root of the tree (i.e., in LUCA) as presence probabilities (PPs): the PP is the probability that the gene was present in LUCA, assuming the model is true. In reality, the model is clearly not consummate, and - particularly when reconstructing ancient events - a cautious and critical approach to interpreting the results of the analyses is essential. Three limitations of our approach are worth bearing in mind: first, the reconstruction is incomplete: some gene families that really were present in LUCA are difficult to map because the signal in their individual gene trees is weak, or because they have since experienced very extensive gene loss or transfer. Second, some gene families with high PPs were not in LUCA; even if the PPs are unbiased, analysing thousands of gene families statistically will result in some false positives. Finally, we note that if a gene originated on the archaeal or bacterial stem, and was then transferred to the other stem prior to the radiation of Archaea and Bacteria, it will appear to map to LUCA in any phylogenetic analysis.

We also note that for several families, inference of gene trees and reconciliations was intractable due to the large number of sequences (>1600 extant tips). These were: K01990, K02003, K00059, K02035, K06147, K00626, K02033, K02034. The vast majority of the sequences placed into clusters that failed were due to having a very small number of sequences, (≤), or a triple COG category as the most frequent one ascribed for that gene family.

Spore photoproduct lyase
One line of evidence for a surface ecology for LUCA comes from the presence of spore photoproduct lyase: (K03716/COG1533: PP: 0.88). In extant prokaryotes, this enzyme repairs methylene-bridged thymine dimers occurring in spore DNA as a result of damage induced through ultraviolet radiation[1,2]. However, it is important to note that this gene family is also found across modern taxa which neither form endospores, nor dwell in environments where they are likely to accrue UV damage to their DNA. For example, it is found in Helarchaeota, which live exclusively in marine sediments. Consequently, it is plausible that the ancestral version of this enzyme might have played a role in DNA repair, although the relevance of high UV levels to this function is ambiguous.

Nitrogen metabolism
Nitrogen metabolism was more variable across the different thresholds, nitrogen fixation seems unlikely, with only our lower threshold inferring any of the pathways to be present (33% pathwise completeness, with the presence of nifH (0.63) and none of the required enzymes being present at > 0.75 PP. However, we did find evidence for dissimilatory nitrate reduction (nitrite reductase, 0.77) across all our confidence thresholds and sampling requirements, we also found a complete assimilatory nitrate reduction pathway inferred to be present at PP > 0.50. We also found support for nitrite reductase, (K00362: 0.77PP)

implicated in dissimilatory nitrite reduction, perhaps for anaerobic respiration. However, given that the other subunits required are not present with high support, the case for the presence of the dissimilatory pathway is not compelling.

Sulfate reduction

We infer assimilatory sulfate reduction to be likely present in LUCA with a 50% completeness at the PP=0.75 threshold (including sulfate adenylyltransferase and sulfite reductase) and 83% completeness at the PP=0.5 threshold, with the addition of phosphoadenosine phosphosulfate reductase to the set.

Ribosomal proteins

We infer 52 ribosomal proteins present at PP ≥ 0.5 and 34 at PP ≥ 0.75; assuming that gene families must be present in at least 1% of sampled prokaryotes at the domain level, the numbers drop to 19 (at PP ≥ 0.5) and 9 (at ≥ 0.75).

Aminoacytl-tRNA synthetases

We found strong support (PP > 0.75) for the aminoacyl-tRNA synthetases: Arg, Asn, Gly, Ile, Leu, Lys, Phe, Trp, Tyr and Val. At our lower threshold, we find support for: Asp, Thre, His, Glu, Cys and Gln. We also find a range of support for 37 partially complete amino acid biosynthetic pathways (see Supplementary Data 1).

Nucleic acids

We find support at all levels for partially complete synthesis or degradation pathways leading to adenine, guanine, cytosine and uracil in LUCA. We find support for thymidine synthesis but not thymine.

DNA replication

LUCA was capable of carrying out DNA replication (enzymes with the sampling requirement: K03471, rnhC, (0.84PP); K10747, LIG1, (0.84PP); K02335, polA (0.76PP); K02347, polX (0.61PP) — without the sampling requirement: K02322, polC (0.99PP); K02338, dnaN (0.89PP); K04800, rfcL (0.89PP); K02319, pol (0.84PP); K02323, polB (0.68PP); K03050, rpoE2 (0.54PP)) and transcription (with the sampling requirement: K10844, ERCC2, (0.97PP) — without the sampling requirement (K13643, iscR (0.98PP); K03120, TBP (0.96PP); K03040, rpoA (0.95PP); K03049, rpoE1 (0.93PP); K03007, RPABC5 (0.90PP); K03044, rpoB1 (0.93PP); K03041, rpoA1 (0.84PP); K03090, sigB (0.74PP); K03046, rpoC (0.69PP)). In addition to the ribosomal proteins mentioned above, we also find evidence for a large number of amino acid and nucleotide synthesis pathways (Supplementary Data 1). Thus, while the results are consistent with the view that these core pathways were already present in LUCA, the reconstruction is likely incomplete.

Cytochrome

While one subfamily of the cytochrome c oxidase subunit I+III family (0.69PP) (i.e.K15408) was inferred to be in the root with a PP of 0.69, all the other five subfamilies are absent. We find, however, that this is likely to be an artefact/false positive based on the clustering of the KO gene families (spread over multiple KEGG KOs).

Hydrogenases

Functional classification of hydrogenases is notoriously difficult, and in many cases additional genome context is needed to be certain about the specific function of particular hydrogenase gene families. One issue we faced in our analyses is the clustering of the key subunits of hydrogenases across different databases, for example, the large subunit I of NiFe hydrogenase is mapped to COG0374, whereas based on our annotation, we found hits with this COG spanning multiple KEGG gene families. When clustered into COG families, we find COG0374 was inferred to be present at the root (PP = 0.96). Analysis of the sequences mapped to this family with hyddb[3] suggested the majority (371/392) were NiFe Hydrogenases. Most (211/392) were inferred to be within the bidirectional (group 3), and of these, the majority (116/211) were placed inside group 3c (Heterodisulfide reductase-linked), and 54 were placed in group 3b (NADP-coupled). If we focus on the more granular KEGG annotations: K06281 (0.90) where 111/142 are group 1 (Respiratory H2-uptake [NiFe] hydrogenases), another gene family: K14126 (PP = 0.92, which also is associated with another COG, COG3259), 116/154 are group 3c, 35/154 are group 3b. The majority of these hydrogenases are capable in at least the hydrogen uptake direction, and although our results would seem to indicate bidirectional hydrogen usage, it is impossible to be certain due to the difficulty to cluster these genes families accurately and the lack of gene cluster information and genomic context. Yet, we find support for other indicators for hydrogenase-associated subunits in the form of small subunits hyaA (0.86), echC (0.91), echE (0.93), mvhD (0.97), mvhG (0.93), hndC (0.94), HndD (0.81); maturation factors: HypF (0.96); expression proteins, HypC (0.87), HypD (0.88), HypE (0.99).

Reverse Gyrase

The evolutionary history of reverse gyrase is complex, involving a gene-fusion event and multiple deep horizontal gene transfer events, and some analyses suggest this gene family likely originated after LUCA, on either the bacterial or archaeal stem[4]. These complexities are difficult to tease out in the context of a systematic, high-throughput approach, so we also explored other options for inferring the temperature preference of LUCA. To do so, we used a previously established classifier marker selection[5] as an alternative for inferring the optimal growth temperature for LUCA. However, these results were similarly ambiguous (thermophile classifiers mean PP: 0.52; psychrophile classifiers mean PP: 0.19; p: 0.007628 Wilcoxon test), and so our analysis does not definitely establish the growth temperature of LUCA.

Topology I

In comparison to topology II, the gene families mapped to LUCA were similar (Pearson's correlation, *r:* 0.6720275, p < 2.2e-16; Spearman's rank, ρ = 0.773, p < 2.2e-16; Wilcoxon signed rank test: p < 2.2e-16), with topology I set of reconciliations being the less conservative of the two analyses and specific pathways. In terms of the qualitative picture of LUCA, reconstructions under both topologies were also very similar, although in general pathways were more filled out under the the topology I inference. For example, 25-36 (at PP ≥0.5 and ≥0.75 thresholds, respectively) components of the glycolysis/gluconeogenesis pathway were mapped to LUCA under topology I, compared to 11-19 under topology II. Reconciliations performed on the maximum likelihood topology (topology I, Extended Data Figure 3) suggest that at the highest threshold of confidence (PP: ≥0.75) LUCA possessed 1087 KEGG gene families, and 1871 at the lower threshold (PP: ≥0.50). By comparison, 682 (PP: ≥0.75) and 1124 (PP: ≥0.50) KEGG families were mapped to LUCA under topology II.

As we inferred with topology II (Extended Data Figure 4), this inferred LUCA was also a fully-fledged prokaryote, not unlike modern Bacteria and Archaea, utilising the Wood-Ljungdahl, Acetyl-CoA, and TCA cycle pathways as an acetogenic and anaerobic organism. The topology I LUCA also possessed the ATP synthase and genes for protein synthesis (25-51 ribosomal proteins), nucleotide and amino acid biosynthesis. Although we find the presence of spore photolyase (see above), the difference in species topology lead to an inferred lack of reverse gyrase.

Genome size estimations

We repeated the analysis for LUCA's genome size and number of proteins (in the main text, this was done using KEGG gene families) for our COG-based gene families with a predictive model trained on the number of the COG families encoded by each of the 700 prokaryotic genomes, the, genome sizes and the number of proteins encoded by these genomes. The number of proteins inferred in LUCA (95% HPD), lower bound: 1466, upper bound: 1739, Mean: 1605. Genome size (95% HPD), lower bound: 1.45Mb, upper bound: 1.72Mb, mean: 1.56Mb.

Fossil calibrations

We used previously established dates for four fossil calibrations and included nine additional calibrations with justifications provided below. Note that five of the newly defined calibrations (i.e., total group Eukarya for the archaeal lineage, Embryophyta, Archaeplastida, and Eumetazoa) have been revised to follow the latest geochronological updates and literature as of December 2023, and so differ from those used in previous studies[6–8]. Note that the root of a phylogeny requires constraining in molecular clock analyses. We constrained the root (representing the duplication of pre-LUCA paralogues) with a hard upper bound equal to the age of the moon forming impact (see below).

LUCA | hard 3347 - hard 4520 Ma

Fossil taxon and specimen: Strelley Pool Formation, Pilbara Craton, Following the justification outlined in Betts et al.[6]

Phylogenetic justification: Following Mahendrarajah et al.[8]

Minimum Age justification: Following Mahendrarajah et al.[8]

Hard maximum age justification: Following Mahendrarajah et al.[8]

Total-group Oxyphotobacteria | hard 2945 - hard 4520 Ma

Fossil taxon and specimen: stable Fe and U-Th-Pb isotopes in the Manzimnyama Banded Ironstone Formation, Fig Tree Group, Barberton, South Africa[9]

Phylogenetic justification: This equates to the traditional concept of Cyanobacteria, excluding Melainabacteria[10], but including the stem to this remaining clade.

Hard Minimum Age: 2945 Ma

Hard Maximum Age: 4520 Ma

Discussion: there are claims and counterclaims for life in the Hadean and Archaean and while we accept that there is credible fossil, sedimentologic and isotopic evidence for life by 3347 Ma[6], there has been insufficient consideration of whether these records evidence the establishment of the crown clade of life - i.e. divergence from LUCA. Implicitly or explicitly, most records have been attributed to the crown clade of life based on claims, direct or indirect, of oxygenic cyanobacteria, either as microfossils, sedimentary structures such as MISS or stromatolites, or Banded Ironstones. The existence of stromatolites would appear to

provide evidence of phototactic bacteria, but they might otherwise represent microbial organisms competing for other nutrients within the water column[11]. Similarly, Banded Ironstone Formations (BIFs) can be formed by reaction with oxygen from abiogenic sources like photolysis[12], hence, BIFs only become a significant proxy for life when they occur in volume (and even then they are not necessarily linked to oxygenic photosynthesis). Betts et al.[6] based their hard minimum age based on low δ^{56}Fe, interpreted to indicate quantitative Fe(II) oxidation by O_2, from the Manzimnyama BIF in the Fig Tree Group, Barberton, South Africa[9,13], the minimum age of which is constrained maximally by a spherule layer dated to 3258 Ma ± 3 Myr[14], and minimally by an overlying volcanic unit dated to 3226 Ma ± 1 Myr[15]. However, both Satkoski et al.[9] and Wang et al.[13] analysed δ^{56}Fe of bulk BIF which contains $FeCO_3$ that has lower δ^{56}Fe and higher Mn/Fe than Fe oxides. Furthermore, both anoxygenic photoferrotrophs and abiotic UV oxidation can drive quantitative Fe(II) oxidation and Busigny et al.[16] obtained contrasting results (high δ^{56}Fe) for the same deposit, concluded that anoxygenic photoferrotrophs account for partial Fe(II) oxidation.
Accumulation and preservation of Mn oxides is a more reliable fingerprint for O_2 oases; anaerobic Mn oxidation is possible[17] but it is the preservation of the geochemical or isotopic signal that is important given that oxidised species such as Mn (IV) would quickly revert back to Mn (II) under anoxic conditions and so the oxidation signal would not be preserved in the rock record. Mn oxidation has been evidenced by low δ^{98}Mo in the Mozaan Group[18,19], Pongola Supergroup, South Africa and dated to 2954 Ma ±9 Myr based on U-Pb dating of an Andesite in the uppermost layer of the Mozaan Group[20]. This is compatible with evidence of Ce oxidation anomalies which are interpreted to reflect Mn oxidation[21].
Hard minimum age justification: Mn oxidation has been evidenced by low δ^{98}Mo in the Mozaan Group[18,19], Pongola Supergroup, South Africa and dated to 2954 Ma ±9 Myr based on U-Pb dating of an Andesite in the uppermost layer of the Mozaan Group[20]. Thus, our minimum constraint is 2945 Ma.
Hard maximum age justification: The Moon forming impact would have effectively sterilised the Earth and so it serves as an effective basis for establishing a hard maximum age constraint. Pb-Pb dating carried out on Moon rocks, yielding a date of 4.51 Ga ± 10 Myr [22] a date which has also recently been confirmed by reanalysis of the Apollo mission zircons [23]. Thus, our maximum constraint is 4.52 Ga.

Crown Oxyphotobacteria | *Gloeobacter-Pseudanabaena*: soft 2013.6 - soft 3448 Ma
Fossil taxon and specimen: *Eoentophysalis belcherensis* (Holotype) GSC type no. 42770, from the upper part of Kasegalik Formation, Belcher Supergroup, following Mahendrarajah et al.[8]
Phylogenetic justification: Following Mahendrarajah et al.[8]
Minimum Age justification: Following Mahendrarajah et al.[8]
Soft maximum age justification: Following Mahendrarajah et al.[8]

Total-group Eukaryota (Archaeal lineage) | hard 1619.1 - hard 3448 Ma
Fossil taxon and specimen: Changzhougou Formation, North China, following the justification outlined in Betts et al.[6].
Hard Minimum Age justification: The minimum age of the Changzhougou Formation is established based on dated ashes in the overlying Chuanlinggou Formation, dated to 1625.3 ± 6.2 Myr [24], thus 1619.1 Ma. Fossils found there have a cytoskeleton, a cyst with a complex wall and excystment mechanisms.

Hard maximum age justification: This based on the age of the Strelley Pool Formation, which includes the oldest unequivocal evidence of cellular life, and substantially older than any vaguely credible record of a eukaryote grade organism. The maximum depositional age of the Strelley Pool Formation has been established based on SIMS and LA–ICPMS U–Pb dating of detrital zircons as 3414 ± 34 Ma[25], affording a maximum age constraint of 3448 Ma.

Total group Eukaryota (Mitochondrial lineage) | soft 1085.57 - hard 4520 Ma
Fossil taxon and specimen: *Simia sp.* (Fig. 2D[26]) from core WPB4-105 m in the Nonesuch Shale, Oronto Group, at the Keweenaw Peninsula of Upper Peninsula, Michigan, USA[26,27]
Phylogenetic justification: Fossil euglenids have been known for some time[28] and the veracity of Silurian records has been tested based on ultrastructural evidence [29]. The oldest possible record is Simia sp. which has been interpreted based on its similarity to cysts of extant freshwater and estuarine euglenids[26,27,30].
Soft Minimum Age justification: The minimum age of the Nonesuch Formation is not well constrained but the underlying Copper Harbor Conglomerates interdigitates with Lake Shore Traps which have been dated to 1085.57 Ma ± 0.25 Myr[31]. The lower part of the Nonesuch Formation has been dated to 1078 Ma ± 24 Myr[32] but this does not establish a minimum age. Therefore, we use the age from the underlying Lake Shore Traps to establish a soft minimum bound on this record of Simia sp.
Soft maximum age justification: The Moon forming impact would have effectively sterilised the Earth and so it serves as an effective basis for establishing a hard maximum age constraint on LUCA. Pb-Pb dating carried out on Moon rocks, yielding a date of 4.51 Ga ± 10 Myr[22] a date which has also recently been confirmed by reanalysis of the Apollo mission zircons[23]. Thus, our maximum constraint is 4.52 Ga.

Crown-group Eukaryota (LECA) | soft 1085.57- soft 1879.6 Ma
Fossil taxon and specimen: *Simia* sp. (Fig. 2D[26]) from core WPB4-105 m in the Nonesuch Shale, Oronto Group, at the Keweenaw Peninsula of Upper Peninsula, Michigan, USA[26,33].
Phylogenetic justification: Fossil euglenids have been known for some time[28] and the veracity of Silurian records has been tested based on ultrastructural evidence[29]. The oldest possible record is *Simia* sp. which has been interpreted based on its similarity to cysts of extant freshwater and estuarine euglenids[26,29,30].
Soft Minimum age justification: The minimum age of the Nonesuch Formation is not well constrained but the underlying Copper Harbor Conglomerates interdigitates with Lake Shore Traps which have been dated to 1085.57 Ma ± 0.25 Myr[31]. The lower part of the Nonesuch Formation has been dated to 1078 Ma ± 24 Myr[32] but this does not establish a minimum age. Therefore, we use the age from the underlying Lake Shore Traps to establish a soft minimum bound on this record of *Simia* sp.
Soft maximum age justification: Following Mahendrarajah et al.[8]

Dikarya | *Agaricus-Batrachochytrium*: hard 392.1 - soft 1891 Ma
Fossil taxon and specimen: *Paleopyrenomycites devonicus*, Rhynie, Aberdeenshire, Scotland. Lower Devonian, following Betts et al.[6]
Phylogenetic justification: Following Betts et al.[6]
Minimum Age justification: Following Betts et al.[6]
Soft Maximum Age Justification: Following Betts et al.[6]

Metazoa | hard 574 - soft 609 Ma
Fossil taxon and specimen: Charnia masoni (OUM ÁT.429/p) from level DRK-10 within the Drook Formation (sensu Matthews et al.[34]) at Mistaken Point, Newfoundland[35]
Phylogenetic justification: Following Yu et al.[36]
Minimum Age justification: Following Yu et al.[36]
Soft Maximum Age Justification: Following Yu et al.[36]

Eumetazoa | hard 561.1 - soft 590.8 Ma
Fossil taxon and specimen: *Auroralumina attenboroughii* (GSM 106119; British Geological Survey, Nottingham, UK) from Bed B, Bradgate Formation, Maplewell Group, Charnian Supergroup, Leicestershire, UK[37].
Phylogenetic justification: Following Yu et al.[36]
Minimum justification: Following Yu et al.[36]
Soft maximum justification: Following Yu et al.[36], Weng'an

Crown Foraminifera | *Elphidium-Reticulomyxa*: hard 522.5 - hard 1891 Ma
Fossil taxon and specimen: *Platysolenites cooperi* from the Chapel Island Formation, Newfoundland, Canada, Lower Cambrian, following Betts et al.[6]
Phylogenetic justification: Following Betts et al.[6]
Minimum Age justification: Following Betts et al.[6]
Soft Maximum Age Justification: Following Betts et al.[6]

Crown Archaeplastida / Crown plastids | hard 1030 - hard 1879.6 Ma
Specimen and fossil taxon: *Bangiomorpha pubescens*. (Holotype) HUPC 62912, Slide HUST-1A, England Finder coordinates: O-35. Locality and Stratigraphy level: Lower Hunting Formation, Somerset Island, Arctic Canada.
Hard Minimum age: 1030 Ma (1047 Ma +13/–17 Myr [38])
Hard maximum: is based on the maximum age interpretation of the Gunflint Chert, a diverse and well-documented microbiota that is widely interpreted to be absent of eukaryotes. This has been dated to 1878.3 Ma ± 1.3 Myr[39], yielding a maximum age of 1879.6 Ma.

Embryophyta: Bryophyta – Tracheophyta | hard 430.54 - soft 515.5 Ma
Fossil taxon and specimen: *Cooksonia barrandei* (D 552a,b, National Museum, Prague) from the Motol Formation at Loděnice, Špičatý vrch-Barrandovy Jámy, Czech Republic [40].
Phylogenetic justification: Dichotomously branching axes with terminal sporangia containing trilete spores, all of which are total-group tracheophyte characters at the least[41].
Minimum Age justification: the holotype was recovered from *Monograptus belophorus* Biozone, Motol Formation, Middle Sheinwoodian, Wenlock, Silurian. The top of the M. belophorus Biozone is dated to 430.54 Ma[42].
Maximum age justification: A soft maximum age constraint for crown group Embryophyta must encompass all total group Embryophyta, including all cryptospores (sensu stricto[43]). The oldest deposits to yield probable non-marine palynomorphs include the Rogersville Shale and Pumpkin Valley Shale of the Conasauga Group of east Tennessee[44], and the Bright Angel Shale of the Tonto Group of Arizona[43,45], correlated to the Glossopleura and Ehmaniella Laurentian trilobite Biozones, dated to Stage 5 of Series 3, the oldest part of the Mid Cambrian[46]. Cryptospores have recently been reported from the Rome Formation[46] that underlies the Conasauga Group and is correlated to the *Olenellus* trilobite Biozone of Stage

4 of Series 2, the latest part of the Early Cambrian. The base of the Olenellus Biozone is dated to approximately 515.5 Ma[47].

Mesangiospermae: *Arabidopsis-Oryza* | hard 125 - soft 247.2 Ma[7]
Fossil taxon and specimen: Tricolpate pollen grain [palynological sample BRN 126], from the middle Atherfield Wealden Bed 35 of the Cowleaze Chine Member, Vectis Formation, Barremian (Early Cretaceous), of the Isle of Wight, following Morris et al.[7]
Phylogenetic justification: Following Morris et al.[7]
Minimum Age justification: Following Morris et al.[7]
Soft Maximum Age Justification: Following Morris et al.[7]

References

1. Chandor, A. *et al.* Dinucleotide spore photoproduct, a minimal substrate of the DNA repair spore photoproduct lyase enzyme from Bacillus subtilis. *J. Biol. Chem.* **281**, 26922–26931 (2006).
2. Chandra, T. *et al.* Spore photoproduct lyase catalyzes specific repair of the 5R but not the 5S spore photoproduct. *J. Am. Chem. Soc.* **131**, 2420–2421 (2009).
3. Søndergaard, D., Pedersen, C. N. S. & Greening, C. HydDB: A web tool for hydrogenase classification and analysis. *Sci. Rep.* **6**, 34212 (2016).
4. Catchpole, R. J. & Forterre, P. The Evolution of Reverse Gyrase Suggests a Nonhyperthermophilic Last Universal Common Ancestor. *Mol. Biol. Evol.* **36**, 2737–2747 (2019).
5. Jensen, D. B., Vesth, T. C., Hallin, P. F., Pedersen, A. G. & Ussery, D. W. Bayesian prediction of bacterial growth temperature range based on genome sequences. *BMC Genomics* **13 Suppl 7**, S3 (2012).
6. Betts, H. C. *et al.* Integrated genomic and fossil evidence illuminates life's early evolution and eukaryote origin. *Nat Ecol Evol* **2**, 1556–1562 (2018).
7. Morris, J. L. *et al.* The timescale of early land plant evolution. *Proc. Natl. Acad. Sci. U. S. A.* **115**, E2274–E2283 (2018).
8. Mahendrarajah, T. A. *et al.* ATP synthase evolution on a cross-braced dated tree of life. *Nat. Commun.* **14**, 7456 (2023).
9. Satkoski, A. M., Beukes, N. J., Li, W., Beard, B. L. & Johnson, C. M. A redox-stratified

ocean 3.2 billion years ago. *Earth Planet. Sci. Lett.* **430**, 43–53 (2015).

10. Shih, P. M., Hemp, J., Ward, L. M., Matzke, N. J. & Fischer, W. W. Crown group Oxyphotobacteria postdate the rise of oxygen. *Geobiology* **15**, 19–29 (2017).
11. Bosak, T., Knoll, A. H. & Petroff, A. P. The Meaning of Stromatolites. *Annu. Rev. Earth Planet. Sci.* **41**, 21–44 (2013).
12. Cairns-Smith, A. G. Precambrian solution photochemistry, inverse segregation, and banded iron formations. *Nature* **276**, 807–808 (1978).
13. Wang, C. *et al.* Archean to early Paleoproterozoic iron formations document a transition in iron oxidation mechanisms. *Geochim. Cosmochim. Acta* **343**, 286–303 (2023).
14. Byerly, G. R., Kröner, A., Lowe, D. R., Todt, W. & Walsh, M. M. Prolonged magmatism and time constraints for sediment deposition in the early Archean Barberton greenstone belt: evidence from the Upper Onverwacht and Fig Tree groups. *Precambrian Res.* **78**, 125–138 (1996).
15. Kamo, S. L. & Davis, D. W. Reassessment of Archean crustal development in the Barberton Mountain Land, South Africa, based on U-Pb dating. *Tectonics* **13**, 167–192 (1994).
16. Busigny, V. *et al.* Iron and sulfur isotope constraints on redox conditions associated with the 3.2Ga barite deposits of the Mapepe Formation (Barberton Greenstone Belt, South Africa). *Geochim. Cosmochim. Acta* **210**, 247–266 (2017).
17. Daye, M. *et al.* Light-driven anaerobic microbial oxidation of manganese. *Nature* **576**, 311–314 (2019).
18. Planavsky, N. J. *et al.* Evidence for oxygenic photosynthesis half a billion years before the Great Oxidation Event. *Nat. Geosci.* **7**, 283–286 (2014).
19. Ossa, F. O. *et al.* Limited oxygen production in the Mesoarchean ocean. *Proceedings of the National Academy of Sciences* **116**, 6647–6652 (2019).
20. Mukasa, S. B., Wilson, A. H. & Young, K. R. Geochronological constraints on the magmatic and tectonic development of the Pongola Supergroup (Central Region), South Africa. *Precambrian Res.* **224**, 268–286 (2013).

21. Zhang, K. & Shields, G. A. Sedimentary Ce anomalies: Secular change and implications for paleoenvironmental evolution. *Earth-Sci. Rev.* **229**, 104015 (2022).
22. Hanan, B. B. & Tilton, G. R. 60025: relict of primitive lunar crust? *Earth and Planetary Science Letters* vol. 84 15–21 Preprint at https://doi.org/10.1016/0012-821x(87)90171-3 (1987).
23. Barboni, M. *et al.* Early formation of the Moon 4.51 billion years ago. *Sci Adv* **3**, e1602365 (2017).
24. Li, H. *et al.* Recent advances in the study of the Mesoproterozoic geochronology in the North China Craton. *Journal of Asian Earth Sciences* vol. 72 216–227 Preprint at https://doi.org/10.1016/j.jseaes.2013.02.020 (2013).
25. Gardiner, N. J., Wacey, D., Kirkland, C. L., Johnson, T. E. & Jeon, H. Zircon U–Pb, Lu–Hf and O isotopes from the 3414 Ma Strelley Pool Formation, East Pilbara Terrane, and the Palaeoarchaean emergence of a cryptic cratonic core. *Precambrian Res.* **321**, 64–84 (2019).
26. Wellman, C. H. & Strother, P. K. The terrestrial biota prior to the origin of land plants (embryophytes): a review of the evidence. *Palaeontology* **58**, 601–627 (2015).
27. The Nonesuch Formation Lagerstätte: a rare window into freshwater life one billion years ago. *Journal of the Geological Society* https://www.lyellcollection.org/doi/abs/10.1144/jgs2020-133.
28. Gray, J. & Boucot, A. J. Is Moyeria a euglenoid? *Lethaia* **22**, 447–456 (1989).
29. Strother, P. K., Taylor, W. A., van de Schootbrugge, B., Leander, B. S. & Wellman, C. H. Pellicle ultrastructure demonstrates that Moyeria is a fossil euglenid. *Palynology* **44**, 461–471 (2020).
30. Hindák, F., Wolowski, K. & Hindáková, A. Cysts and their formation in some neustonic Euglena species. *Annales de Limnologie - International Journal of Limnology* **36**, 83–93 (2000).
31. Fairchild, L. M., Swanson-Hysell, N. L., Ramezani, J., Sprain, C. J. & Bowring, S. A. The end of Midcontinent Rift magmatism and the paleogeography of Laurentia. *Lithosphere*

9, 117–133 (2017).

32. Cumming, V. M., Poulton, S. W., Rooney, A. D. & Selby, D. Anoxia in the terrestrial environment during the late Mesoproterozoic. *Geology* **41**, 583–586 (2013).
33. Strother, P. K., and Wellman, C. H. The Nonesuch Formation Lagerstätte: a rare window into freshwater life one billion years ago. *J. Geol. Soc. London* **178**, (2020).
34. Matthews, J. J. *et al.* A Chronostratigraphic Framework for the Rise of the Ediacaran Macrobiota: New Constraints from Mistaken Point Ecological Reserve, Newfoundland. *GSA Bulletin* **133**, 612–624 (2021).
35. Liu, A. G., McIlroy, D., Matthews, J. J., and Brasier, M. D. A new assemblage of juvenile Ediacaran fronds from the Drook Formation, Newfoundland. *Journal of the Geological Society* **169**, 395–403 (2012).
36. Yu, D. *et al.* Hagfish genome elucidates vertebrate whole-genome duplication events and their evolutionary consequences. *Nat Ecol Evol* (2024) doi:10.1038/s41559-023-02299-z.
37. Dunn, F. S. *et al.* A crown-group cnidarian from the Ediacaran of Charnwood Forest, UK. *Nat Ecol Evol* **6**, 1095–1104 (2022).
38. Gibson, T. M. *et al.* Precise age of Bangiomorpha pubescens dates the origin of eukaryotic photosynthesis. *Geology* vol. 46 135–138 Preprint at https://doi.org/10.1130/g39829.1 (2018).
39. Fralick, P., Davis, D. W. & Kissin, S. A. The age of the Gunflint Formation, Ontario, Canada: single zircon U‑Pb age determinations from reworked volcanic ash. *Canadian Journal of Earth Sciences* vol. 39 1085–1091 Preprint at https://doi.org/10.1139/e02-028 (2002).
40. Libertín, M., Kvaček, J., Bek, J., Žárský, V. & Štorch, P. Sporophytes of polysporangiate land plants from the early Silurian period may have been photosynthetically autonomous. *Nat Plants* **4**, 269–271 (2018).
41. Clarke, J. T., Warnock, R. C. M. & Donoghue, P. C. J. Establishing a time-scale for plant evolution. *New Phytol.* **192**, 266–301 (2011).

42. Melchin, M. J., Sadler, P. M. & Cramer, B. D. The Silurian period. in *Geologic Time Scale 2020* (eds. Gradstein, F. M., Ogg, J. G., Schmitz, M. D. & Ogg, G. M.) 695–732 (Elsevier, 2020).
43. Strother, P. K., Wood, G. D., Taylor, W. A., and Beck, J. H. Middle Cambrian cryptospores and the origin of land plants. *Memoirs of the Association of Australasian Palaeontologists* **29**, 99–113.
44. Strother, P. K. & Beck, J. H. Spore-like microfossils from Middle Cambrian strata: expanding the meaning of the term cryptospore. in *Pollen and spores: morphology and biology* (eds. Harley, M. M., Morton, C. M. & Blackmore, S.) 413–424 (Kew Publishing, Royal Botanic Gardens, Kew, 2000).
45. Baldwin, C. T., Strother, P., Beck, J., and Rose, E. Palaeoecology of the Bright Angel Shale in the eastern Grand Canyon, Arizona, USA, incorporating sedimentological, ichnological and palynological data. *Geological Society, London, Special Publications* **228**, 213–236.
46. Strother, P. K. Systematics and evolutionary significance of some new cryptospores from the Cambrian of eastern Tennessee, USA. *Rev. Palaeobot. Palynol.* **227**, 28–41 (2016).
47. Peng, S. C., Babcock, L. E. & Ahlberg, P. Chapter 19 - The Cambrian Period. in *Geologic Time Scale 2020* (eds. Gradstein, F. M., Ogg, J. G., Schmitz, M. D. & Ogg, G. M.) 565–629 (Elsevier, 2020).
48. Eren, A. M. *et al.* Anvi'o: an advanced analysis and visualization platform for 'omics data. *PeerJ* **3**, e1319 (2015).
49. Ciccarelli, F. D. *et al.* Toward automatic reconstruction of a highly resolved tree of life. *Science* **311**, 1283–1287 (2006).
50. Zaremba-Niedzwiedzka, K. *et al.* Asgard archaea illuminate the origin of eukaryotic cellular complexity. *Nature* **541**, 353–358 (2017).
51. Yutin, N., Makarova, K. S., Mekhedov, S. L., Wolf, Y. I. & Koonin, E. V. The deep archaeal roots of eukaryotes. *Mol. Biol. Evol.* **25**, 1619–1630 (2008).

52. Harris, J. K., Kelley, S. T., Spiegelman, G. B. & Pace, N. R. The genetic core of the universal ancestor. *Genome Res.* **13**, 407–412 (2003).
53. Petitjean, C., Deschamps, P., López-García, P. & Moreira, D. Rooting the domain archaea by phylogenomic analysis supports the foundation of the new kingdom Proteoarchaeota. *Genome Biol. Evol.* **7**, 191–204 (2014).
54. Williams, T. A., Cox, C. J., Foster, P. G., Szöllősi, G. J. & Embley, T. M. Phylogenomics provides robust support for a two-domains tree of life. *Nat Ecol Evol* **4**, 138–147 (2020).
55. Rinke, C. *et al.* A standardized archaeal taxonomy for the Genome Taxonomy Database. *Nat Microbiol* **6**, 946–959 (2021).
56. Parks, D. H. *et al.* Selection of representative genomes for 24,706 bacterial and archaeal species clusters provide a complete genome-based taxonomy. *bioRxiv* 771964 (2019) doi:10.1101/771964.
57. Coleman, G. A. *et al.* A rooted phylogeny resolves early bacterial evolution. *Science* **372**, (2021).

Supplementary Figures

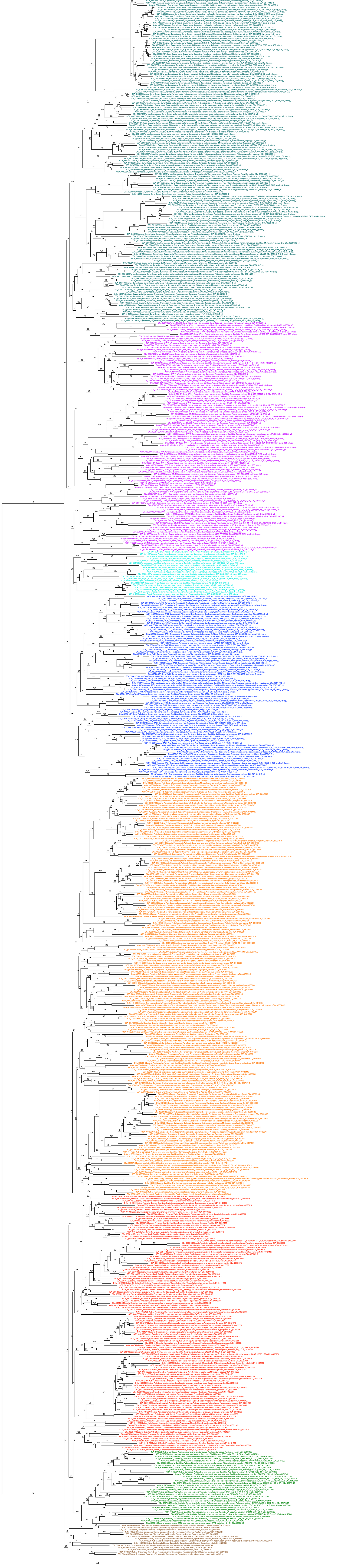

Supplemental Figure 1. Maximum likelihood tree inferred under LG+C20+F+G from a concatenation of 57 single copy orthologues, for the purposes of comparing to constrained topologies with the AU test. Support values are from 10,000 ultrafast bootstraps. The tips are coloured according to taxonomy: Euryarchaeota (teal), DPANN (purple), Asgardarchaeota (cyan), TACK (blue), Gracilicutes (orange), Terrabacteria (red), DST (brown), CPR (green). AU test, P = 0.604, this is a one-sided statistical test.

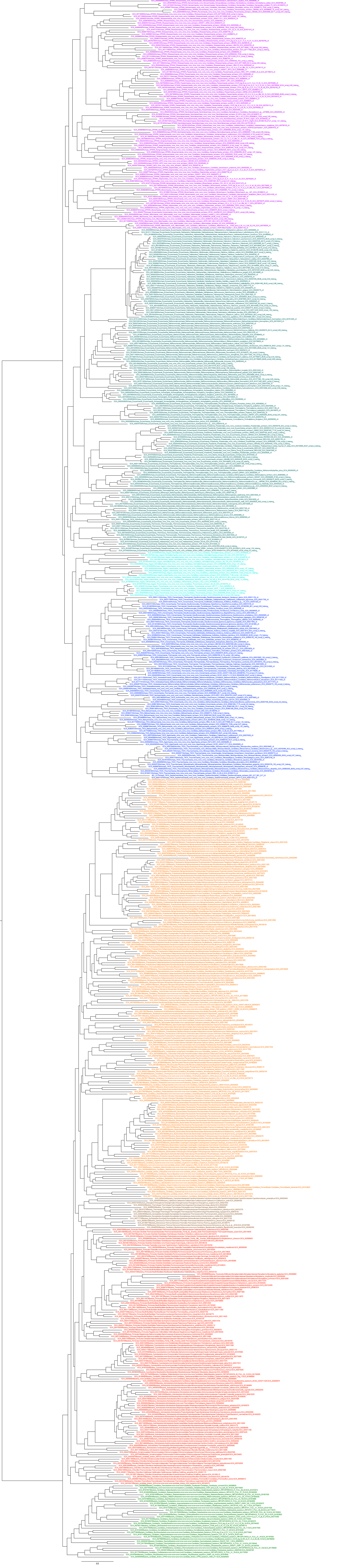

Supplemental Figure 2. Maximum likelihood tree where DPANN is constrained to be sister to all other archaea. Tips coloured according to taxonomy: Euryarchaeota (teal), DPANN (purple), Aasgardarchaeota (cyan), TACK (blue), Gracilicutes (orange), Terrabacteria (red), DST (brown), CPR (green). AU test, P = 0.434, this is a one-sided statistical test.

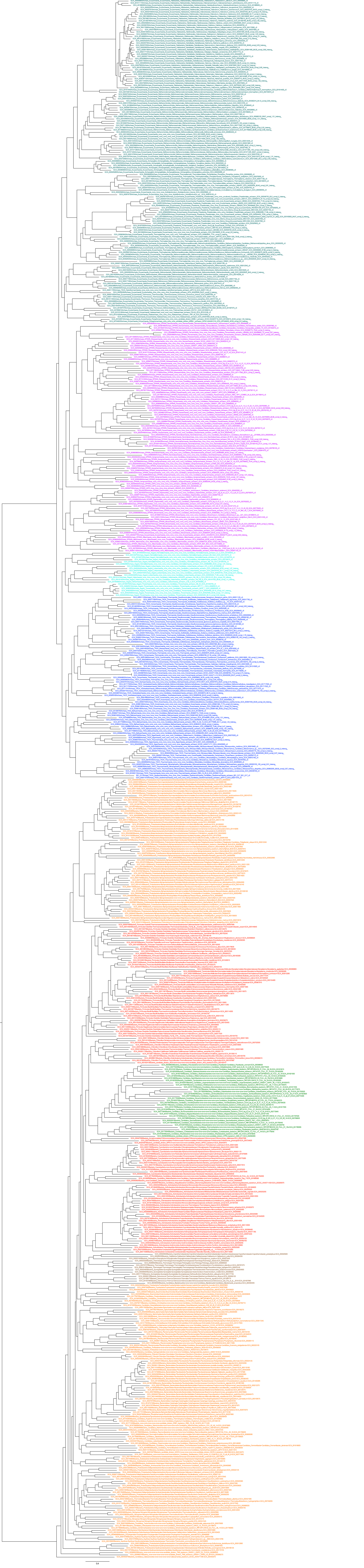

Supplemental Figure 3. Maximum likelihood tree where CPR is constrained to be sister to Chloroflexi. Tips coloured according to taxonomy: Euryarcheota (teal), DPANN (purple), Aasgardarchaeota (cyan), TACK (blue), Gracilicutes (orange), Terrabacteria (red), DST (brown), CPR (green). AU test, P = 0.243, this is a one-sided statistical test.

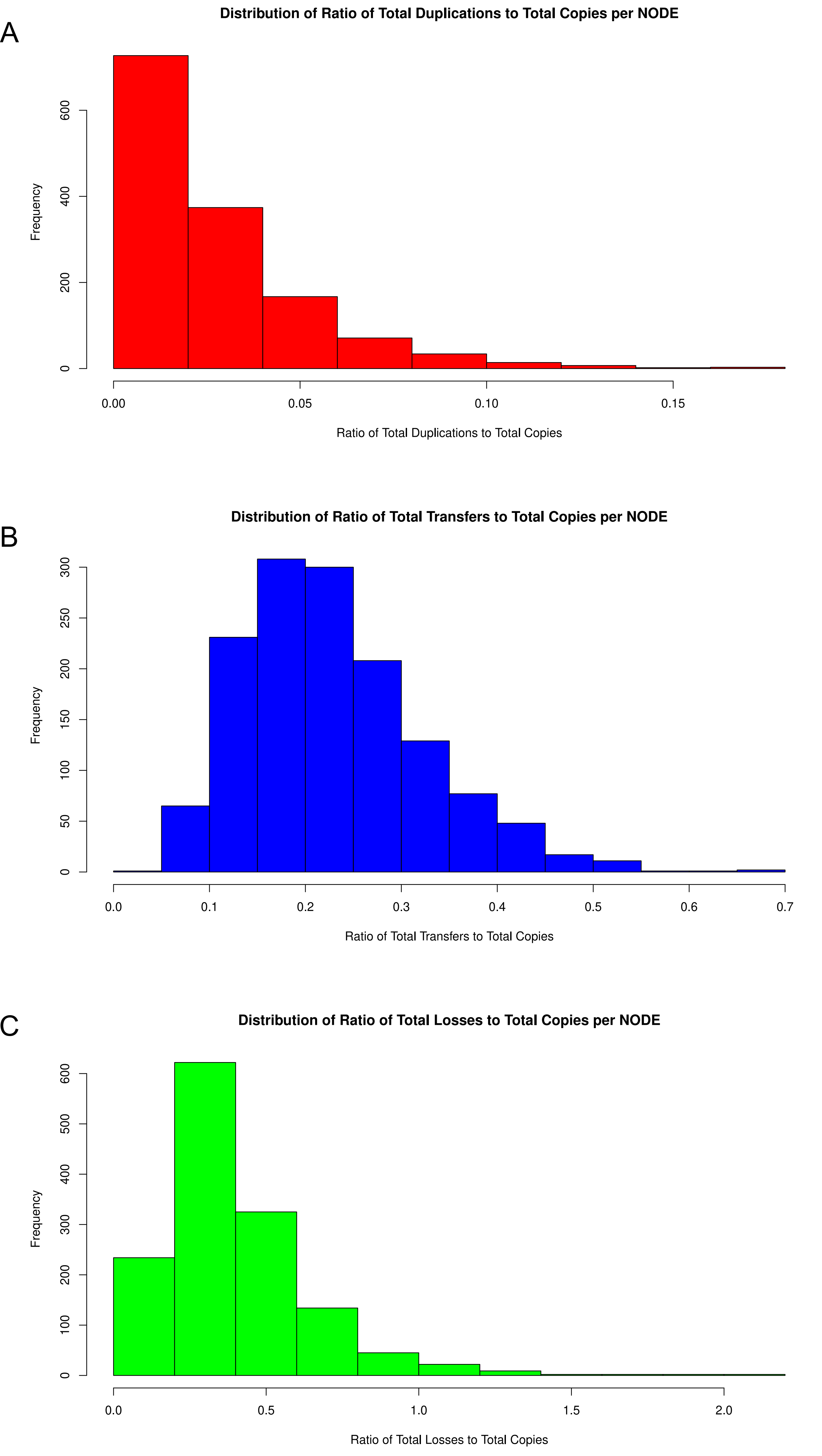
A
Distribution of Ratio of Total Duplications to Total Copies per NODE
Frequency
0
200
400
600
0.00
0.05
0.10
0.15
Ratio of Total Duplications to Total Copies
B
Distribution of Ratio of Total Transfers to Total Copies per NODE
Frequency
0
50
100
150
200
250
300
0.0
0.1
0.2
0.3
0.4
0.5
0.6
0.7
Ratio of Total Transfers to Total Copies
C
Distribution of Ratio of Total Losses to Total Copies per NODE
Frequency
0
100
200
300
400
500
600
0.0
0.5
1.0
1.5
2.0
Ratio of Total Losses to Total Copies

Supplemental Figure 4. A tree with labelled nodes to apply equality constraints (i.e., nodes that are cross-braced). By labelling nodes a and b using the same label (i.e., '#1'), and nodes u and v using the same label (i.e., '#2'), equality constraints are enforced when running the PAML program MCMCtree on the following node ages: $t_a = t_b$, $t_u = t_v$. Black dots indicate nodes which age is constrained by fossil calibrations.

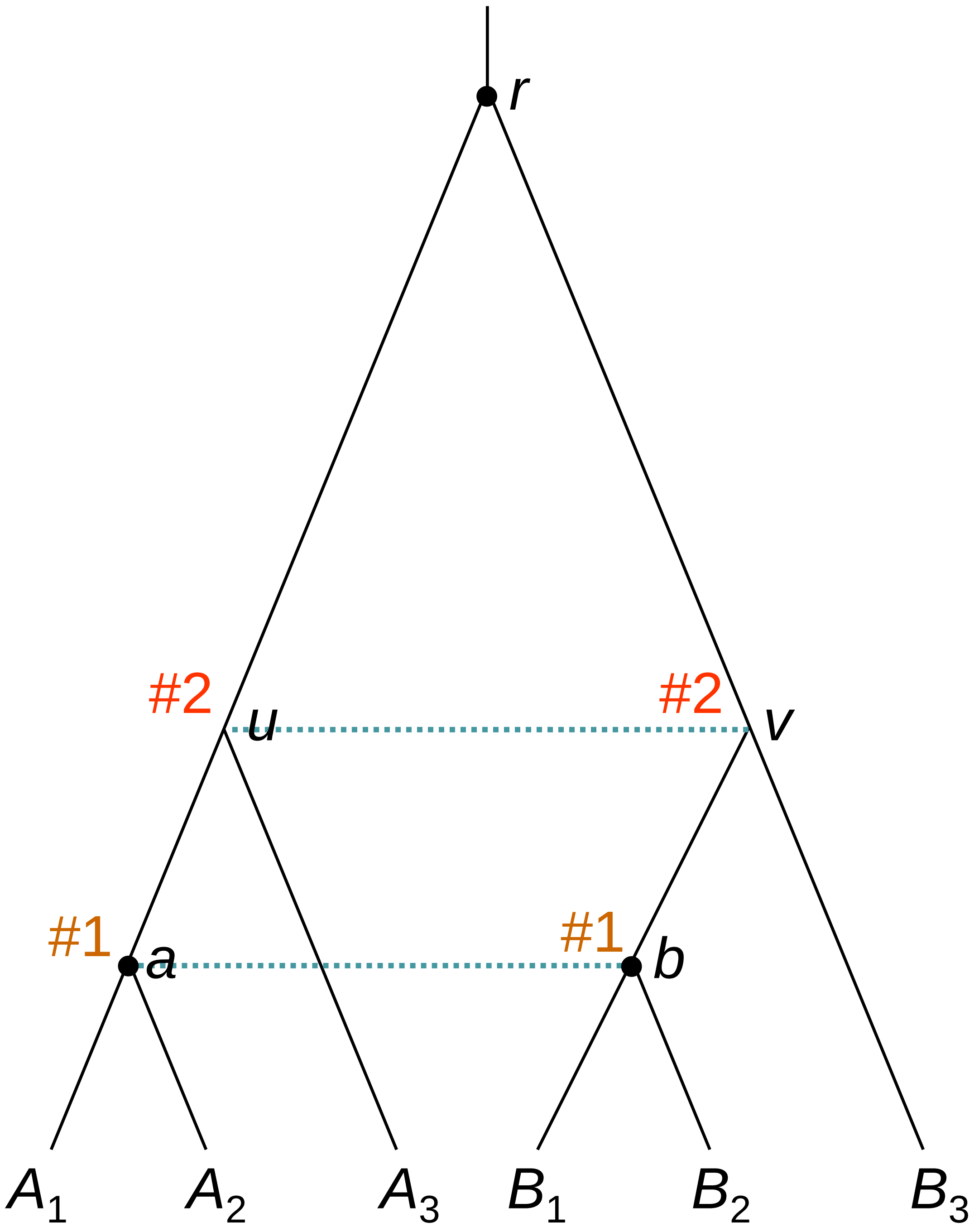

<u>*Fossil constraints*</u>

$0.2 < t_a = t_b < 0.4$

$0.9 < t_r < 1.1$

<u>*Equality (cross-bracing) constraints*</u>

$t_a = t_b,\ t_u = t_v$

Supplemental Figure 5. Plots showing the user-specified prior vs. the effective prior for all the calibrated nodes analysed under "cross-bracing A" in MCMCtree. The prior densities based on the node age constraints we specified based on the fossil record or geological events are referred to as "user-specified priors" (blue). The corresponding prior densities for such nodes built by dating programs when no data are used are the "effective priors" (black), which result from considering the joint prior (i.e., user-specified constraints and other priors incorporated in the analyses).

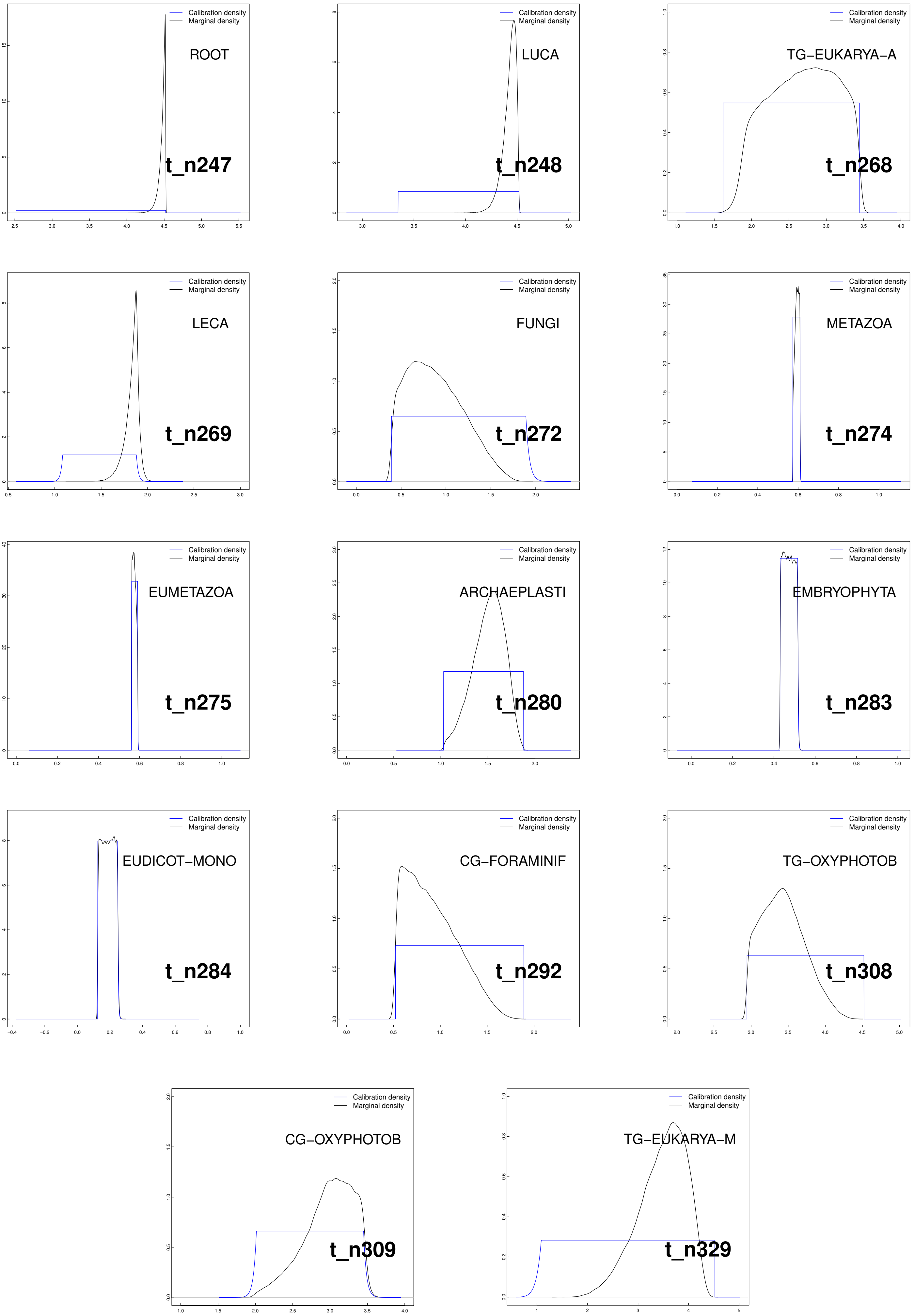
Calibration density
Marginal density
ROOT
t_n247
LUCA
t_n248
TG−EUKARYA−A
t_n268
LECA
t_n269
FUNGI
t_n272
METAZOA
t_n274
EUMETAZOA
t_n275
ARCHAEPLASTI
t_n280
EMBRYOPHYTA
t_n283
EUDICOT−MONO
t_n284
CG−FORAMINIF
t_n292
TG−OXYPHOTOB
t_n308
CG−OXYPHOTOB
t_n309
TG−EUKARYA−M
t_n329

Supplemental Figure 6. Convergence plots for the posterior time estimates inferred under GBM and ILN when analysing the concatenated and partitioned datasets under "cross-bracing A" in MCMCtree. All the chains we ran for these analyses passed our quality control filters during the MCMC diagnostics, and thus we calculated the corresponding mean posterior time estimates for each of them. To generate these convergence plots, we averaged the mean posterior time estimates for the first 8 chains (x axis) and plotted them against the average of the mean posterior time estimates for the last 8 chains (y axis). The resulting convergence plots show that $x \simeq y$, and thus this qualitative check for chain convergence is passed.

A) Concatenated + GBM

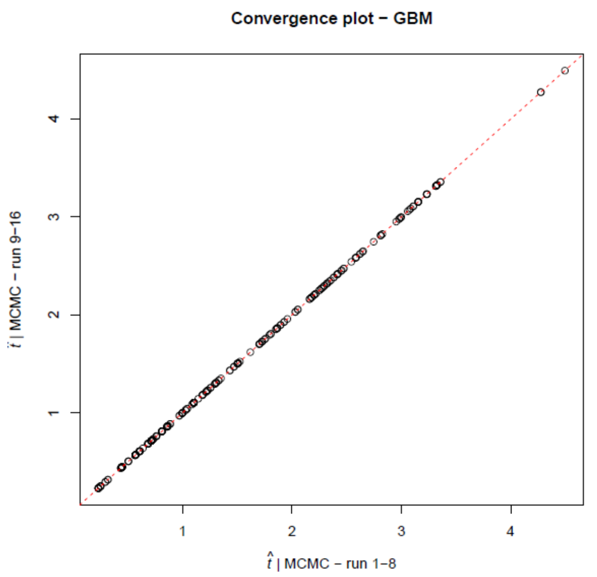

B) Concatenated + ILN

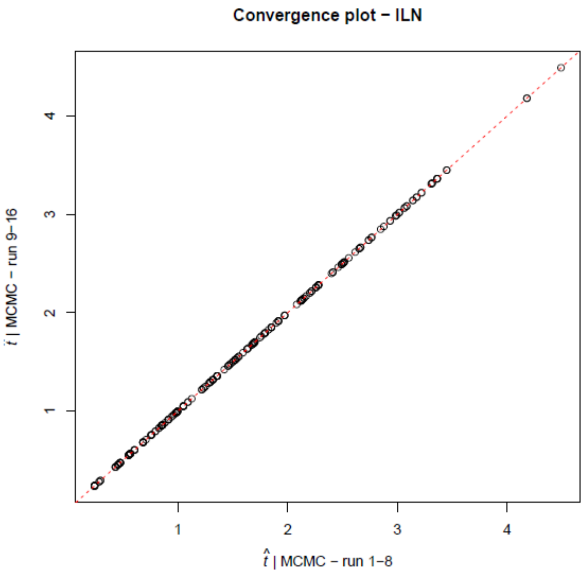

C) Partitioned + GBM

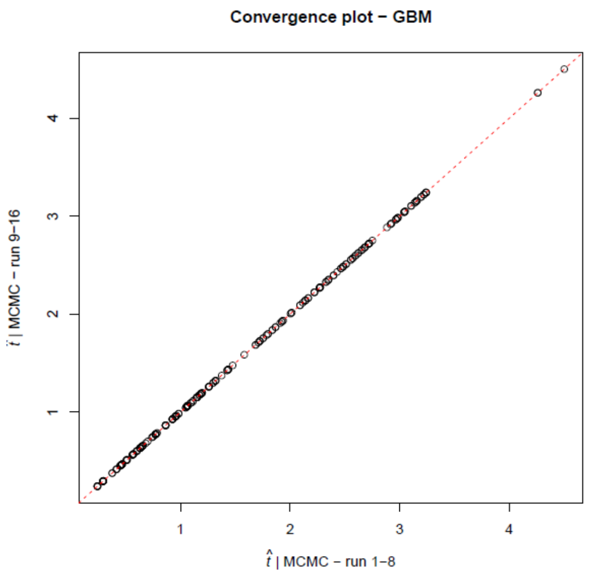

D) Partitioned + ILN

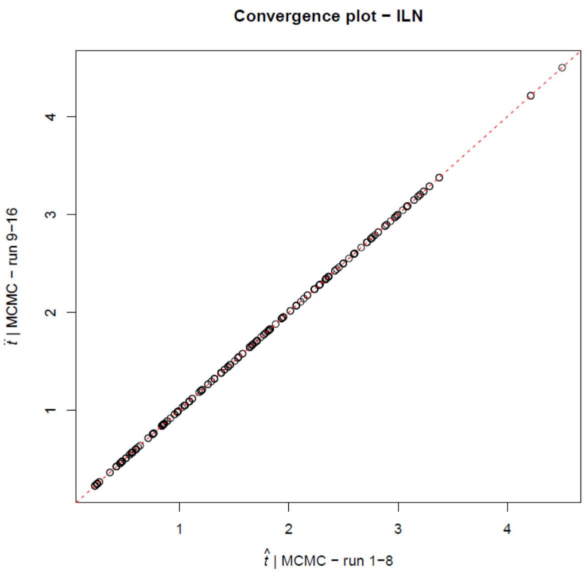

Supplemental Figure 7. Timetree inferred under a Bayesian node-dating approach with cross-bracing A. This figure shows the corresponding posterior time densities of the mirrored nodes for the last universal, archaeal, bacterial, and eukaryotic common ancestors (LUCA, LACA, LBCA and LECA, respectively); as well as the last common ancestor of the mitochondrial lineage (Mito-LECA) and the last plastid-bearing common ancestor (LPCA). Each posterior density corresponds to the posterior time density of each of the analyses we ran under the calibration strategy "cross-bracing A": (blue) partitioned dataset analysed under GBM, (purple) partitioned dataset analysed under ILN, (dark green) concatenated dataset analysed under GBM, (brown) concatenated dataset analysed under ILN. Note that all nodes that correspond to the same divergences were cross-braced during these analyses.

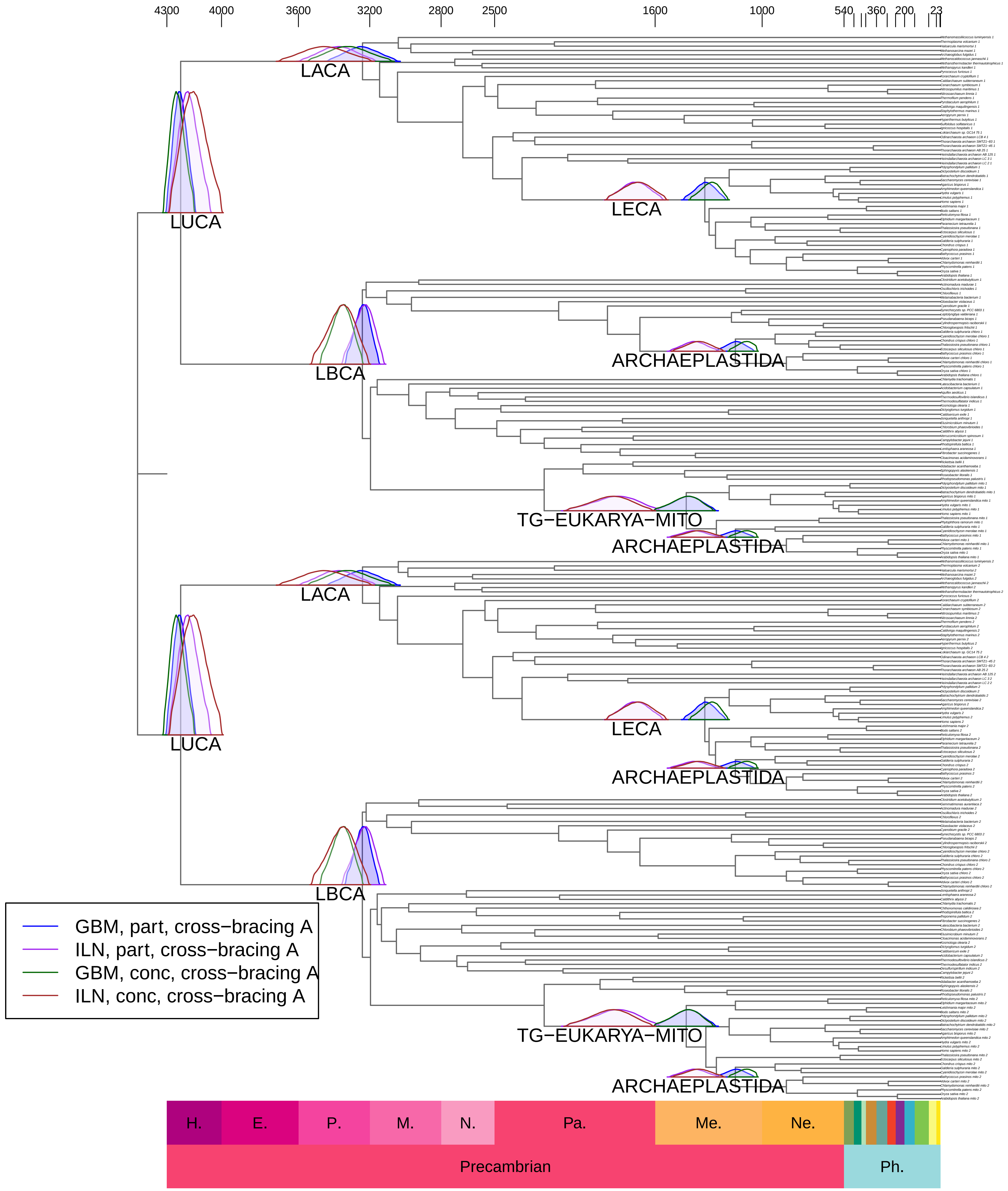
4300
4000
3600
3200
2800
2500
1600
1000
540
360
200
23
LACA
LUCA
LECA
LBCA
ARCHAEPLASTIDA
TG−EUKARYA−MITO
ARCHAEPLASTIDA
LACA
LECA
LUCA
ARCHAEPLASTIDA
LBCA
TG−EUKARYA−MITO
ARCHAEPLASTIDA
GBM, part, cross−bracing A
ILN, part, cross−bracing A
GBM, conc, cross−bracing A
ILN, conc, cross−bracing A
H.
E.
P.
M.
N.
Pa.
Me.
Ne.
Precambrian
Ph.

Supplemental Figure 8. Timetree inferred under a Bayesian node-dating approach with cross-bracing B. This figure shows the corresponding posterior time densities of the mirrored nodes for the last universal, archaeal, bacterial, and eukaryotic common ancestors (LUCA, LACA, LBCA and LECA, respectively); as well as the last common ancestor of the mitochondrial lineage (Mito-LECA) and the last plastid-bearing common ancestor (LPCA). Each posterior density corresponds to the posterior time density of each of the analyses we ran under the calibration strategy "cross-bracing B": (blue) partitioned dataset analysed under GBM, (purple) partitioned dataset analysed under ILN, (dark green) concatenated dataset analysed under GBM, (brown) concatenated dataset analysed under ILN. Note that only nodes that correspond to the same divergences for which there are fossil constraints were cross-braced during these analyses.

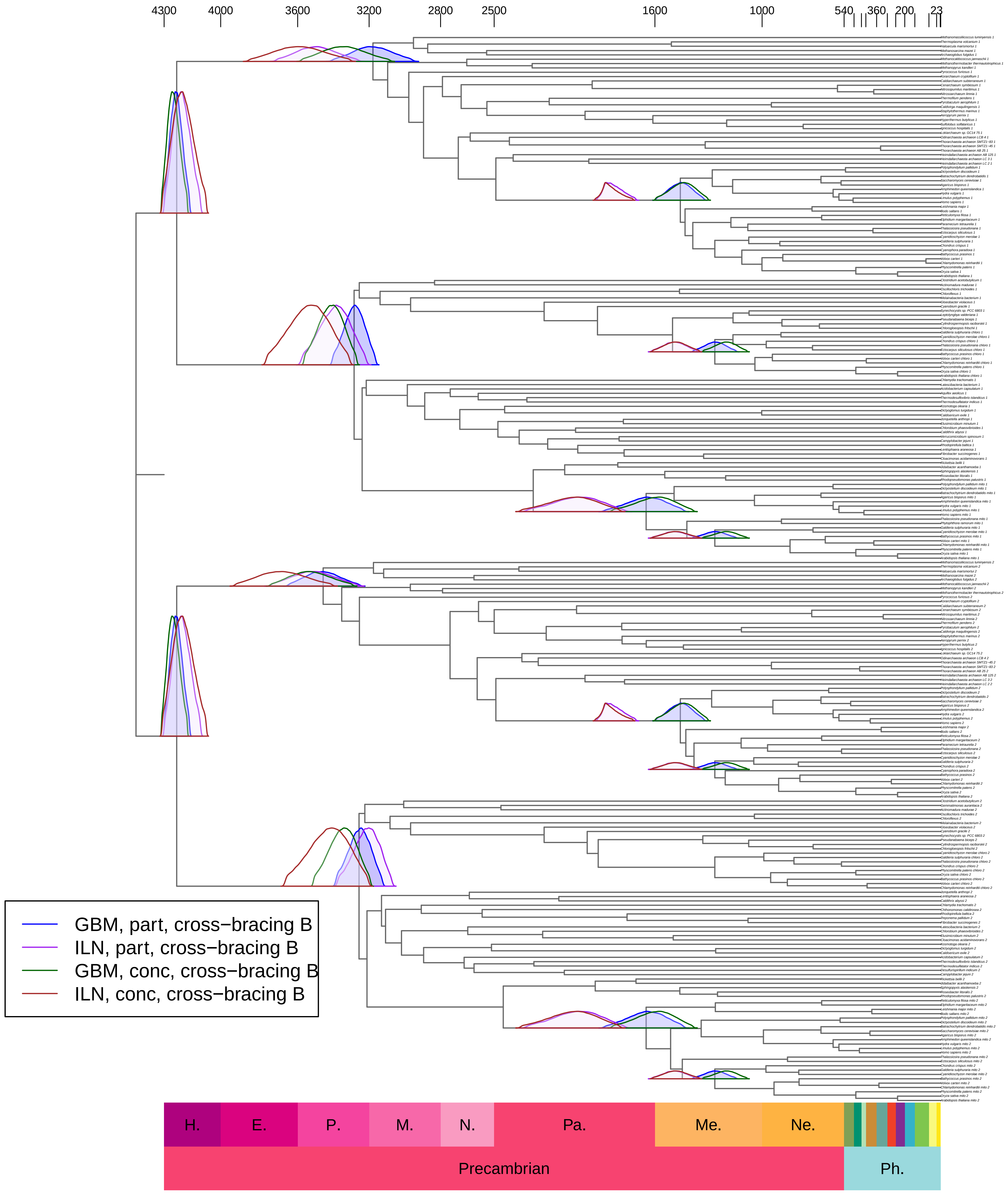
4300
4000
3600
3200
2800
2500
1600
1000
540
360
200
23
GBM, part, cross−bracing B
ILN, part, cross−bracing B
GBM, conc, cross−bracing B
ILN, conc, cross−bracing B
H.
E.
P.
M.
N.
Pa.
Me.
Ne.
Precambrian
Ph.

Supplemental Figure 9. Timetree inferred under a Bayesian node-dating approach without cross-bracing. This figure shows the corresponding posterior time densities of the mirrored nodes for the last universal, archaeal, bacterial, and eukaryotic common ancestors (LUCA, LACA, LBCA and LECA, respectively); as well as the last common ancestor of the mitochondrial lineage (Mito-LECA) and the last plastid-bearing common ancestor (LPCA). Each posterior density corresponds to the posterior time density of each of the analyses we ran when cross-bracing was not enabled: (blue) partitioned dataset analysed under GBM, (purple) partitioned dataset analysed under ILN, (dark green) concatenated dataset analysed under GBM, (brown) concatenated dataset analysed under ILN. Note that we applied the same age constraint to those nodes that corresponded to the same speciation event. Given that such nodes are no longer cross-braced, the posterior time densities therefore differ.

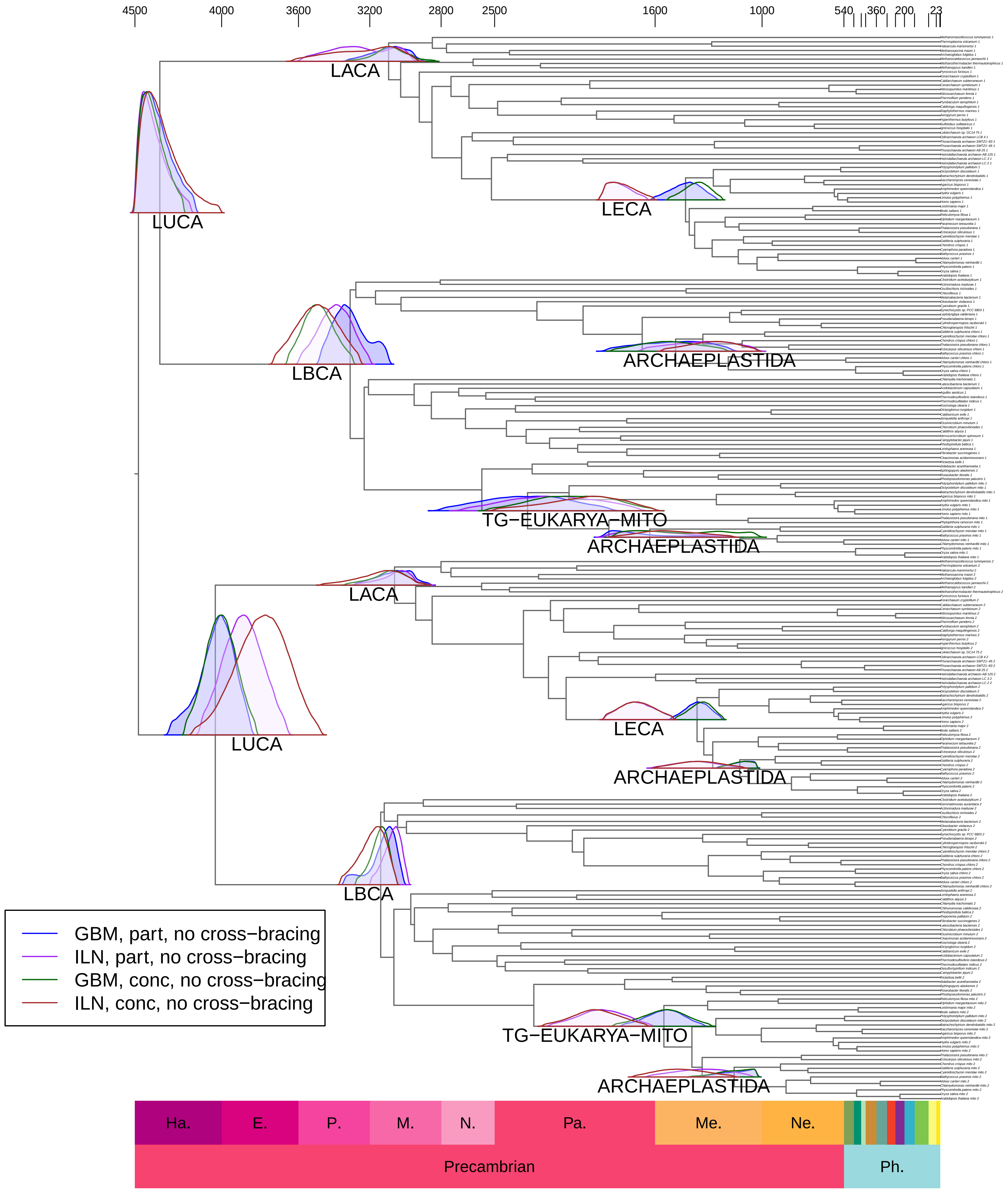

4500
4000
3600
3200
2800
2500
1600
1000
540
360
200
23
LACA
LUCA
LECA
LBCA
ARCHAEPLASTIDA
TG−EUKARYA−MITO
ARCHAEPLASTIDA
LACA
LUCA
LECA
ARCHAEPLASTIDA
LBCA
TG−EUKARYA−MITO
ARCHAEPLASTIDA
GBM, part, no cross−bracing
ILN, part, no cross−bracing
GBM, conc, no cross−bracing
ILN, conc, no cross−bracing
Ha.
E.
P.
M.
N.
Pa.
Me.
Ne.
Precambrian
Ph.

Supplemental Figure 10. Histograms showing the ratio of distribution of duplications, transfers and losses for each node in the species tree (including tips) across KEGG gene families. A) Duplications. B) Transfers C) Losses

Supplementary Data

***Supplementary Data 1.** This table contains the results of the reconciliations for each gene family, KEGG_ko is the KEGG orthology ID, arc_domain_prop is the proportion of the sampled archaea, bac_domain_prop is the proportion of the sampled bacteria, Gene gene name, description and enzyme code, Map refers to the different KEGG maps this KEGG gene family is a component of, Pathway is a text description of the metabolic pathways these gene is a component of, alignment_length refers to the length of the alignment in amino acids, highest_COG_cat refers to the number of sequence placed in the most frequent COG category, difference_1st_and_2nd is the difference between the most frequent COG category and the second most frequent COG category, categories is the number of different COG categories assigned to this KEGG gene family, COG_freq is the proportion of the sequences placed in the most frequent COG category, COG_cat the most frequent COG functional category, archaea is the number of archaeal sequences sampled in the gene family, bacteria is the number of bacterial sequences sampled in the gene family, alternative_COGs is the number of alternative COG gene families assigned across this KEGG orthologous gene family, COG_perc is the proportion of the most frequent COG ID assigned to this KEGG gene family, COG is the COG ID of the most frequenty COG assigned to this gene family, COG_NAME is the description of the most frequent COG ID assigned to this gene family, COG_TAG is the symbol associated with the most frequent COG gene familiy, sequences is the total number of sequences assigned to this gene family, Arc_prop is the proportion of archaea which make up this gene family, Bac_prop is the proportion of bacteria which make up this gene family, constrained_median is the median probability (PP) that this gene was present in LUCA from our reconciliation under the focal constrained tree search across the five independent bootstrap distribution reconciliations, ML_median is the median PP of the gene family being present in LUCA with gene tree bootstrap distributions against the maximum likelihood species tree topology across the 15 independent bootstrap distribution reconciliations, MEAN_OF_MEDIANS the mean value across the constrained and maximum likelihood PP results, RANGE_OF_MEDIANS the range of the PPs for the constrained and maximum likelihood topology PPs for LUCA, Probable_and_sampling_threshold_met is our most stringent category of gene families inferred in LUCA with 0.75+ PP and a sampling requirement of 1% met in both Archaea and Bacteria, Possible_and_sampling_threshold_met is a threshold of 0.50+PP and sampling both domains, Probable is simply 0.75+ PP, Possible is 0.50+ PP.*

***Supplementary Data 2.** Presence probability for COGs. This table contains the results for the reconciliations of COG-based gene family clustering against the constrained focal species tree topology, columns are named similarly to Supplementary Data 2, but each row is a different COG family, the column Modal_KEGG_ko refers to the most frequent KEGG gene family a given COG is found in, sequences_in_modal_KEGG refers to the number of sequences in the most frequent KEGG gene family.*

***Supplementary Data 3.** Module completeness. Estimated pathway completeness for KEGG metabolic modules (with a completeness greater than zero, in at least one confidence threshold) using anvio's stepwise pathway completeness[48]. Module_name is the name of the module, module_category is the broader category the module falls into, module_subcategory is a more specific category, and then possible_anvio includes the gene families with a median PP ≥0.50, probable_anvio related to gene families PP ≥0.75, '_ws' refers to the the sampling requirement being met (presence in at least 1% of the sampled Archaea and Bacteria).*

***Supplementary Data 4.** Marker gene metadata for all markers checked during marker gene curation, including the initial 59 single-copy marker genes used in species-tree inference (see Methods). Data includes marker gene set provenance, marker gene name, marker gene description, presence in different marker gene sets [49–57]., and presence in Archaea and Bacteria. When available, marker genes are matched with their arCOG, TIGR, and COG ID and their respective occurrence across different taxonomic sets is quantified.*

***Supplementary Data 5.** The ratio of duplications, transfers and losses in relation to the total number of copies for the deep ancestral nodes: the last universal (LUCA), archaeal (LACA) and bacterial (LBCA) common ancestors, as well as the average (mean) and 95th percentile.*

***Supplementary Data 6.** Spreadsheet containing a list of the estimated divergence times for all timetree inferences carried out and the corresponding results of the MCMC diagnostics. Tabs "Divtimes_GBM-allnodes" and "Divtimes_ILN-allnodes": list of the estimated divergence times (Ma) for all nodes under the 12 inference analyses we ran under GBM and ILN, respectively. Tabs "Divtimes_GBM-highlighted" and "Divtimes_ILN-highlighted": list of the estimated divergence times (Ma) for selected nodes ordered according to their mirrored nodes under the 12 inference analyses we ran under GBM and ILN, respectively. Tabs "MCMCdiagn_prior", "MCMCdiagn_postGBM", "MCMCdiagn_postILN": each tab contains the statistical results of the MCMC diagnostics we ran for each inference analysis. Note that, despite the analyses carried out when sampling from the prior could have only been done three times (i.e., data are not used, and thus only once under each calibration strategy was enough), we repeated them with each dataset regardless. In other words, results for (i) "concatenated + cross-bracing A" and "partitioned + cross-bracing A"; "concatenated + without cross-bracing" and "partitioned + without cross-bracing"; and (iii) "concatenated +cross-bracing B" and "partitioned + cross-bracing B" would be equivalent; respectively. Abbreviations, Tabs 1-4: "part": partitioned dataset; "conc": concatenated dataset; "cb": cross-bracing A; "notcb":*

without cross-bracing; "fosscb": cross-bracing B; "mean_t": mean posterior time estimate; "2.5%q": 2.5% quantile of the posterior time density for a given node; and "97.5%q": 97.5% quantile of the posterior time density for a given node. Tabs 5-7: "Med. num. samples collected per chain": median of the total amount of samples collected per chain; "Min. num. samples collected per chain": minimum number of samples collected per chain; "Max. num. samples collected per chain": minimum number of samples collected per chain; "Num. samples used to calculate stats": number of samples collected by all chains that passed the filters that were used to calculate the tail-ESS, bulk-ESS, and R-hat values; "tail-ESS": we report the median, minimum, and maximum tail-ESS values; all larger than 100 as required for assuming reliable parameter estimates; "bulk-ESS": we report the median, minimum, and maximum bulk-ESS values; all larger than 100 as required for assuming reliable parameter estimates; "R-hat": minimum and maximum values reported, all smaller than 1.05 as required to assume good mixing.

Supplementary Data 7. Spreadsheet containing a list of the posterior time estimates for LUCA obtained under the main calibration strategy cross-bracing A with the concatenated dataset and with the datasets for the three additional sensitivity analyses. The first column "Label" contains the node number for both the driver and mirror nodes for LUCA (the latter includes the term "-dup" in the label). Columns "mean_t", "2.5%q", and "97.5%q" refer to the estimated mean divergence times, and the 2.5%/97.5% quantiles of the posterior time density for the corresponding node.
-Abbreviations. "GBM": Geometric Brownian motion relaxed-clock model; "ILN": Independent-rate log-normal relaxed-clock model; "main-conc": results obtained with the concatenated dataset analysed in our main analyses under cross-bracing A; "ATP/EF/Leu/SRP/Tyr": results obtained when using each gene alignment separately; "noATP/noEF/noLeu/noSRP/noTyr": results obtained when using concatenated alignments without the gene alignment mentioned in the label as per the "leave-one-out" strategy; "main-bsinbv": results obtained with the concatenated dataset analysed in our main analyses when using branch lengths, Hessian, and gradient calculated under a more complex substitution model to infer divergence times.

Supplementary Data 8. Spreadsheet containing a list of the estimated divergence times for all timetree inferences carried out for the sensitivity analyses and the corresponding results for the MCMC diagnostics. Tabs "Divtimes_GBM-allnodes" and "Divtimes_ILN-allnodes": list of the estimated divergence times (Ma) for all nodes under the 11 inference analyses we ran under GBM and ILN when testing the impact on divergence times estimation when (i) analysing each gene alignment individually, (ii) following a "leave-one-out" strategy, and (iii) using the branch lengths, Hessian, and gradient estimated under a more complex model for timetree inference ("bsinBV" approach). Tabs "Divtimes_GBM-highlighted" and "Divtimes_ILN-highlighted": list of the estimated divergence times (Ma) for selected nodes ordered according to their mirrored nodes uwe ran under GBM and ILN for the sensitivity analyses (we also included the results with the main concatenated dataset for reference). Tabs "MCMCdiagn_prior", "MCMCdiagn_postGBM", "MCMCdiagn_postILN": each tab contains the statistical results of the MCMC diagnostics we ran for the sensitivity analyses. Note that, despite the analyses carried out when sampling from the prior could have only been done once for each different tree topology (i.e., data are not used, only topological changes may affect the resulting marginal densities), we ran them with each dataset regardless as part of our pipeline.
-Abbreviations. Tabs 1-4: "main-conc": results obtained with the concatenated dataset analysed in our main analyses under cross-bracing A; "ATP/EF/Leu/SRP/Tyr": results obtained when using each gene alignment separately; "noATP/noEF/noLeu/noSRP/noTyr": results obtained when using concatenated alignments without the gene alignment mentioned in the label as per the "leave-one-out" strategy; "main-bsinbv": results obtained with the concatenated dataset analysed in our main analyses when using branch lengths, Hessian, and gradient calculated under a more complex substitution model to infer divergence times; "mean_t": mean posterior time estimate; "2.5%q": 2.5% quantile of the posterior time density for a given node; and "97.5%q": 97.5% quantile of the posterior time density for a given node. Tabs 5-7: "Med. num. samples collected per chain": median of the total amount of samples collected per chain; "Min. num. samples collected per chain": minimum number of samples collected per chain; "Max. num. samples collected per chain": minimum number of samples collected per chain; "Num. samples used to calculate stats": number of samples collected by all chains that passed the filters that were used to calculate the tail-ESS, bulk-ESS, and R-hat values; "tail-ESS": we report the median, minimum, and maximum tail-ESS values; all larger than 100 as required for assuming reliable parameter estimates; "bulk-ESS": we report the median, minimum, and maximum bulk-ESS values; all larger than 100 as required for assuming reliable parameter estimates; "R-hat": minimum and maximum values reported, all smaller than 1.05 as required to assume good mixing.
